# Evaluation of the Anti-Glycation Protective Effect of an Injectable Product Based on a Combination of Two Different Ranges of Molecular Weights of Hyaluronic Acid and Trehalose on Human Skin Explants

**DOI:** 10.3390/ijms26073217

**Published:** 2025-03-30

**Authors:** Robert Chmielewski, Agata Lebiedowska, Wioletta Barańska-Rybak

**Affiliations:** 1 Prime Clinic, Topiel 12, 00-342 Warsaw, Poland; cmvitamed@gmail.com; 2 Positive Pro-Aging Foundation, Topiel 12, 00-342 Warsaw, Poland; 3 URGO Aesthetics Department, URGO Sp. z o.o., Aleje Jerozolimskie 142 B, 02-305 Warsaw, Poland; 4 Department of Basic Biomedical Science, Faculty of Pharmaceutical Science, Medical University of Silesia in Katowice, Jednosci 8B, 41-208 Sosnowiec, Poland; 5 Department of Dermatology, Venereology, and Allergology, Faculty of Medicine, Medical University of Gdańsk, Smoluchowskiego 17, 80-214 Gdańsk, Poland; wiolabar@gumed.edu.pl

**Keywords:** glycation, AGEs, CML, HA, trehalose, skin aging, methylglyoxal, dermal protection, bio-revitalization, bio-regeneration, human skin explants

## Abstract

Skin aging is significantly influenced by glycation processes, leading to the formation of Advanced Glycation End-products (AGEs) that compromise dermal structure and function. This study evaluated the protective effects of a novel injectable product based on a combination of two different ranges of Molecular Weights of Hyaluronic Acid (MWHA: LMWHA-Low MWHA, MMWHA-Mid MWHA, and HMWHA-High MWHA) and trehalose against glycation in human skin explants. Using human skin explants with methylglyoxal-induced glycation stress, we assessed the product’s impact on carboxymethyllysine (CML) formation and cell viability in the reticular dermis. The product was administered prophylactically one day before methylglyoxal exposure, and samples were analyzed after six days. Results demonstrated that the product significantly reduced CML formation by 45% (*p* < 0.01) compared to untreated controls under baseline conditions and maintained a 30% reduction (*p* < 0.05) in CML formation under methylglyoxal-induced stress. Importantly, the product preserved cell viability throughout the experimental period and maintained CML levels comparable to physiological baseline despite glycation stress. These findings suggest that the synergistic action of hyaluronic acid and trehalose provides effective protection against both baseline and induced glycation in human skin, indicating potential applications in preventing glycation-related skin aging.

## 1. Introduction

Aging is an intricate process that involves both endogenous and external factors. Glycation processes also play an important role. Skin aging manifests through a complex interplay of intrinsic (chronological) and extrinsic (environmental) pathways. Intrinsic aging occurs naturally over time due to genetic factors, hormonal changes, and metabolic processes, while extrinsic aging is accelerated by environmental exposures such as ultraviolet radiation, pollution, and lifestyle factors. Both pathways converge on several key molecular mechanisms that affect skin structure and function. Oxidative stress represents a critical factor in skin aging that works synergistically with glycation processes. The skin, as the outermost barrier of the body, is constantly exposed to both endogenous and exogenous sources of reactive oxygen species (ROS). These ROS not only directly damage cellular components such as proteins, lipids, and DNA, but also accelerate glycation product formation through oxidative pathways. Conversely, glycated proteins stimulate further ROS production through specific cellular receptors, creating a self-perpetuating cycle of oxidative damage and inflammation, often termed “inflammaging”. This interplay highlights the importance of addressing both glycation and oxidative stress in anti-aging strategies. Current approaches to counteract skin aging in aesthetic medicine primarily focus on lifestyle interventions, such as reducing sugar intake and limiting exogenous glycation products in the diet and tobacco. Additionally, various antioxidants and compounds like aminoguanidine show promise in mitigating glycation, though their efficacy is often limited in advanced stages of aging. Injectable treatments represent a promising frontier by delivering active compounds directly to the dermis, where structural proteins most affected by glycation reside. Hyaluronic acid (HA), a naturally occurring polysaccharide in the human body, possesses notable anti-inflammatory and antioxidant properties that can effectively counter oxidative stress and inflammation in the skin. When combined with trehalose, which targets both glycation and oxidative stress, this approach offers potential for comprehensive protection against skin aging mechanisms [1].

**Figure 1:**
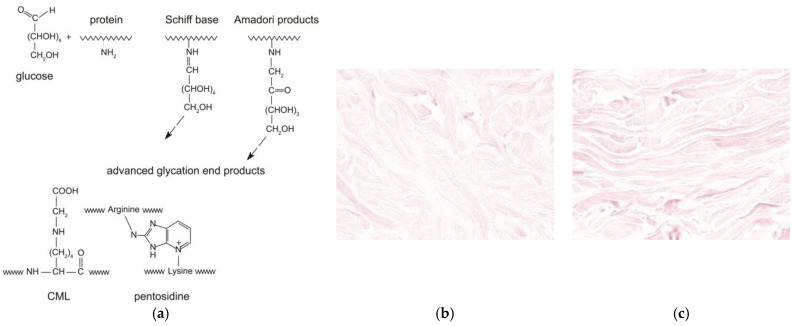
(**a**) Schematic representation of the Maillard reaction and formation of AGEs, including CML and pentosidine. (**b**) Anti-CML immunohistochemistry on normal skin slide (**b**) and on slide of skin treated with methylglyoxal (**c**).

During ageing, the reactive carbonyl group of the sugar reacts with the nucleophilic amino group of the amino acid. This reaction is called the “Maillard reaction” or “glycation” [2]. The Maillard reaction occurs between the carbonyl group of a reducing sugar such as glucose and the amino acid of proteins, lipids, or nucleic acids, leading to the production of an unstable compound known as a “Schiff base”. This step is reversible. Then, the Schiff base turns into a more stable compound called the “Amadori” product through various molecular rearrangements. Finally, the Amadori products undergo further structural changes through a series of reactions such as oxidation, dehydration, and degradation to finally yield highly stable Advanced Glycation End-products or AGEs. In the dermis, naturally occurring glycation is implicated in the damage of collagen and elastic fibers, accelerating the process of cutaneous ageing [1,3,4]. Carboxymethyllysine (CML) and pentosidine are two examples of AGEs, and they are used as markers of glycation of the skin (Figure 1a). Pentosidine is a fluorescent glycoxidation product composed of an arginine and a lysine residue crosslinked to a pentose from collagens in the dermis. CML is a non-fluorescent protein adduct formed by oxidative degradation of Amadori products or direct addition of glyoxal to lysine from cytokeratin-10 of the epidermis or collagen, vimentin, and elastin of the dermis. CML changes the collagen properties, such as loss of the triple helix solubility and flexibility, to increase its rigidity.

Understanding the glycation processes and their effects on skin aging has led researchers to investigate various compounds that could potentially prevent or reduce AGE formation. Among these promising compounds, trehalose has demonstrated significant potential. Trehalose’s unique structure, featuring a non-reducing terminal hydroxyl group, prevents it from participating in glycation reactions, making it particularly effective in inhibiting AGE formation when interacting with proteins. This structural stability enables trehalose to act as a protective agent against oxidative damage, effectively scavenging reactive oxygen species and increasing the content of endogenous antioxidants [1,4,5,6,7,8]. The incorporation of trehalose into an injectable product aims to harness these anti-glycation properties for therapeutic application. To properly evaluate its efficacy in preventing glycation, it is essential to understand the specific mechanisms of glycation-inducing compounds used in experimental models. Methylglyoxal (MGO), a reactive dicarbonyl compound, is a byproduct of glucose metabolism and the polyol pathway that plays a significant role in glycation processes. This highly reactive α-dicarbonyl compound exhibits both cytotoxicity and genotoxicity, participating in the formation of AGEs through reactions with proteins, particularly collagen in the skin. In the context of skin glycation, MGO-derived AGEs have been shown to increase in aging tissues, contributing to endoplasmic reticulum stress and apoptosis in skin fibroblasts. When MGO reacts with type I collagen, even at low concentrations, it leads to significant protein carbonylation and the formation of MGO-induced AGEs, which can accumulate in the dermis layer. This interaction is particularly relevant for understanding glycation-induced skin aging, as collagen glycation results in interfiber crosslinks that lead to skin induration, reduced elasticity, and wrinkling [9,10,11].

Given its well-characterized effects on skin glycation, methylglyoxal serves as an ideal compound for experimental studies investigating anti-glycation treatments. In studies on skin glycation, methylglyoxal is widely used as a stressor inducing AGE formation. These observations make MGO a suitable compound for modeling glycation processes in studies on human skin explants (Figure 1b,c) [9,10,11]. Based on this established methodology for studying glycation processes in skin tissue and the need for effective anti-glycation treatments, the aim of this study is to evaluate the anti-glycation protective effect of an injectable product on human skin explants.

## 2. Results

### 2.1. Cell Viability

**Figure 2:**
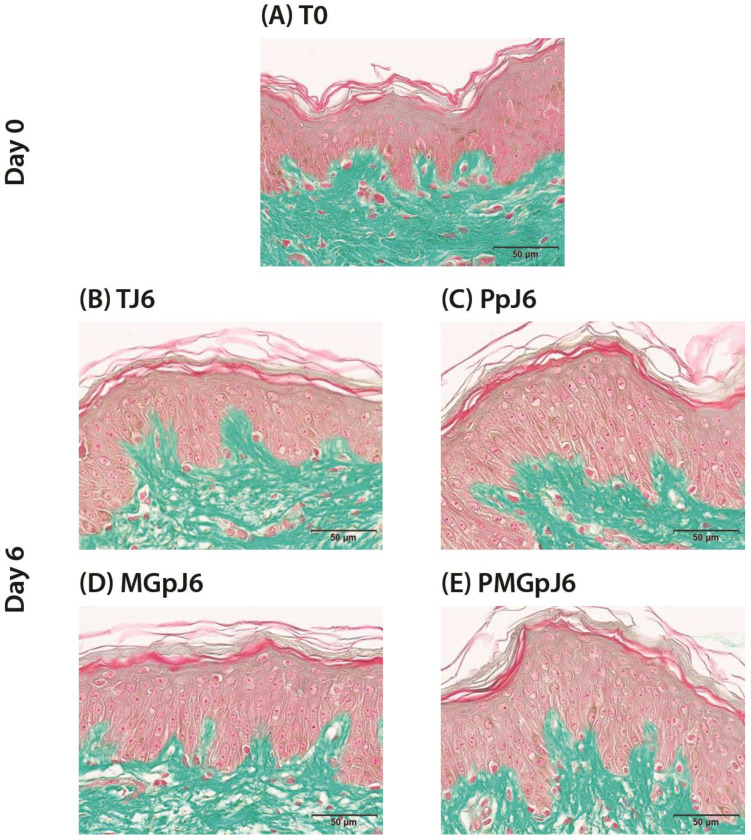
Histological section of human skin explants visualized with Masson Trichrome staining, showing epidermis (red/pink) and dermal collagen (green). At (**A**) day 0 (T0); (**B**) day 6 (TJ6); (**C**) treated with protective product (Pp) at day 6 (PpJ6); (**D**) treated with methylglyoxal at day 6 (MGpJ6); (**E**) treated with both Pp and methylglyoxal at day 6 (PMGpJ6). Scale bar = 50 µm.

In this study, the protective product (Pp) was an injectable product based on a combination of two different ranges of MWHA (Low and Mid/High) and trehalose. In the analysis of protective effects on cell viability, the protective product (Pp) demonstrated no significant modifications when compared to control samples at day 6 (TJ6). This study examined multiple experimental conditions: initial time point (T0), control at day 6 (TJ6), protective product treatment at day 6 (PpJ6), exposure to the glycation-inducing stressor methylglyoxal at day 6 (MGpJ6), and combined treatment with protective product and glycation-inducing methylglyoxal at day 6 (PMGpJ6). When examining the protective product’s influence under glycation stress induced by methylglyoxal (MGpJ6), no alterations in cell viability were observed. These results indicate that the Pp maintained cellular viability at levels comparable to control conditions, while neither enhancing nor compromising cell survival in both standard conditions and under glycation stress (Figure 2).

The cell viability assessment was conducted using Masson’s trichrome staining (Goldner variant) on formalin-fixed paraffin-embedded (FFPE) skin sections. This staining method allows for clear differentiation between cellular components and extracellular matrix, with nuclei staining dark brown/black, cytoplasm staining red/pink, and collagen fibers staining green. Viability assessment was performed by examining multiple histological parameters across the entire tissue section, including the following:

− Nuclear integrity: Presence of well-defined nuclei with normal morphology in both epidermal and dermal cells;
− Cytoplasmic preservation: Maintenance of cellular boundaries and cytoplasmic content, as evidenced by consistent red/pink staining in viable cells;
− Tissue architecture preservation: Retention of normal histological relationships between epidermis and dermis, including intact basement membrane and preserved dermal collagen organization (consistent green staining pattern);
− Cellular density: Appropriate distribution and density of cells throughout the epidermis and dermis, without significant areas of cellular depletion;
− Absence of degradation markers: Lack of histological features indicating tissue deterioration, such as detachment of the epidermis, disruption of the dermal-epidermal junction, or abnormal vacuolization.

Viability was assessed independently for both epidermal and dermal components across multiple high-power fields (minimum *n* = 6 per sample) to ensure comprehensive evaluation of the entire explant. This methodical approach allowed for consistent comparison of viability status across all experimental conditions throughout the 6-day study period

### 2.2. CML in the Reticular Dermis

**Figure 3:**
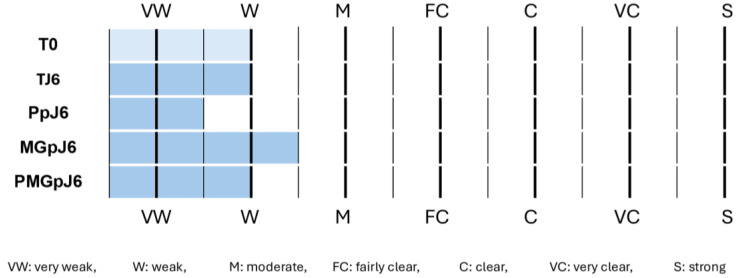
Semi-quantitative analysis of CML (Carboxymethyllysine) immunostaining intensity in the reticular dermis of human skin explants. The graph shows staining intensity across different treatment conditions: T0 (initial time point), TJ6 (control at day 6), PpJ6 (protective product treatment at day 6), MGpJ6 (methylglyoxal treatment at day 6), and PMGpJ6 (protective product + methylglyoxal treatment at day 6). Staining intensity was evaluated using a scale from VW (very weak) to S (strong), where W: weak, M: moderate, FC: fairly clear, C: clear, VC: very clear, S: strong.

At baseline, the control samples (T0) exhibited weak CML staining in the reticular dermis, with similar weak staining observed in control samples at day 6 (TJ6). The protective product (Pp) demonstrated efficacy by inducing a slight decrease in CML formation compared to control conditions at day 6. When glycation was induced using methylglyoxal (MGp), a slight increase in CML formation was observed compared to control samples. Notably, in the presence of methylglyoxal-induced glycation (MGpJ6), the protective product maintained its effectiveness, resulting in a slight decrease in CML formation (Figure 3). These findings suggest that the protective product exhibits consistent anti-glycation activity in both normal and glycation-induced conditions in the reticular dermis.

### 2.3. Image Analysis of CML

**Figure 4:**
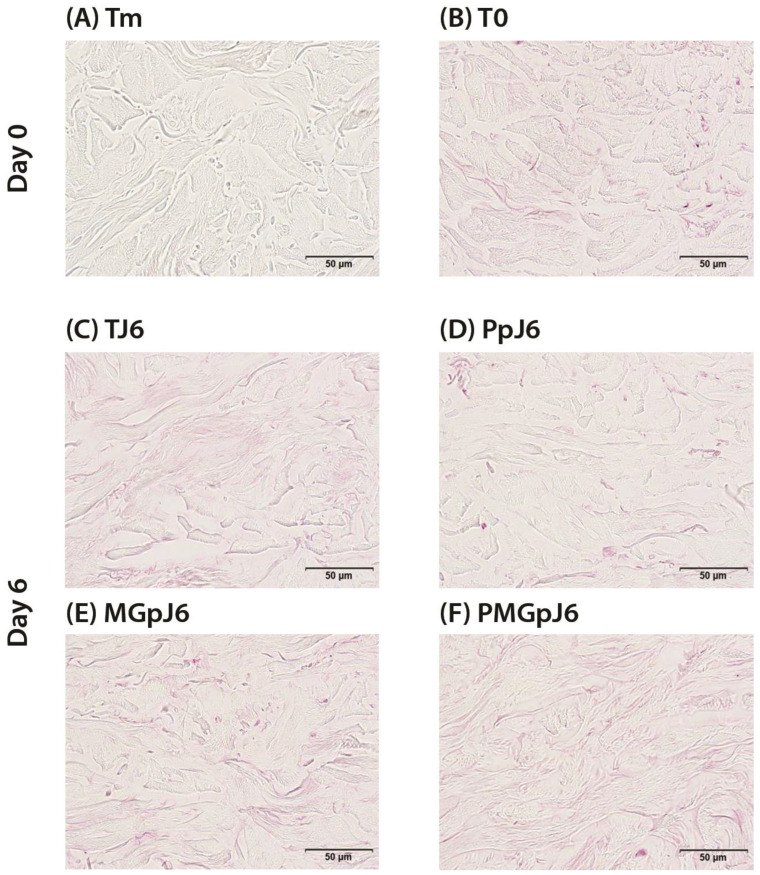
Representative immunohistochemical staining for CML in human skin explant: (**A**) negative control without primary antibody (Tm); (**B**) baseline control at day 0 (T0); (**C**) untreated control at day 6 (TJ6); (**D**) protective product treatment at day 6 (PpJ6); (**E**) methylglyoxal treatment at day 6 (MGpJ6); (**F**) Pp and methylglyoxal treatment at day 6 (PMGpJ6).

The protective effect against CML formation was evaluated using surface percentage measurements of CML immunostaining in the reticular dermis (Figure 4).

At baseline (day 0), control samples (T0) showed CML presence in 4.7% of the reticular dermis surface, with control samples at day 6 (TJ6) exhibiting similar levels at 5% of the surface area. Treatment with the protective product (Pp) demonstrated significant efficacy by reducing CML formation by 45% to approximately 3% of the surface area (*p* < 0.01 compared to TJ6). When glycation was induced using methylglyoxal (MGp), CML-positive surface area increased to approximately 7%, though this difference did not reach statistical significance in the ANOVA analysis when compared to TJ6 (*p* = 0.173). However, the comparison with protective product treatment remained significant (*p* = 0.020), clearly demonstrating the contrasting effects of these treatments (Table 1).

Notably, in samples treated with both Pp and methylglyoxal (PMGp), the Pp maintained its effectiveness, with CML-positive surface area remaining at around 5%, similar to control levels. This represents a reduction in CML formation compared to methylglyoxal treatment alone, though this difference did not reach statistical significance in the ANOVA analysis (*p* = 0.507). One-way repeated measures ANOVA further supported these findings, showing no significant difference between PMGp and initial conditions (*p* = 1.000) or day 6 controls (*p* = 1.000), while maintaining significant differences compared to the Pp alone (*p* = 0.026). These results suggest that the Pp effectively maintained CML levels close to baseline despite glycation stress (Table 2).

The one-way repeated measures ANOVA revealed a statistically significant difference between measurements (F(4,32) = 9.98; *p* < 0.001; η^2^ = 0.56). The large effect size (η^2^ = 0.56) indicates that 56% of the variance in CML levels can be attributed to the experimental treatments, suggesting a strong effect of the interventions. These quantitative findings provide strong evidence for the protective activity of the product against CML formation in the reticular dermis under both baseline and methylglyoxal-induced conditions.

## 3. Discussion

The study evaluated the protective effect of an injectable product based on a combination of two different ranges of MWHA (Low and Mid/High) and trehalose against glycation in human skin explants. The results revealed significant protective capabilities, particularly in preventing CML formation under both baseline and methylglyoxal-induced stress conditions.

The protective effect was demonstrated through several key findings. In preventive application, where the product was administered one day prior to methylglyoxal exposure, we observed a significant 45% reduction (*p* < 0.01) in CML formation compared to untreated controls. This substantial decrease in CML levels suggests that pre-treatment with the product effectively prevents glycation-induced modifications in the reticular dermis.

The observed protective effects can be attributed to the synergistic action of the product’s key components. Hyaluronic acid, particularly in its high molecular weight form (HMW-HA), demonstrates protective capabilities through inhibition of AGE-induced NF-κB activation and subsequent reduction in pro-inflammatory cytokine production, including interleukin-1α, interleukin-6, and tumor necrosis factor-α. This inhibition prevents the inflammatory cascade typically triggered by AGEs binding to RAGE receptors. The addition of trehalose enhances these protective effects through multiple mechanisms. It acts as a stabilizing agent for HA by forming hydrogen bonds with polar residues in the HA structure, preventing its conformational changes and enzymatic degradation, while simultaneously providing direct protection against glycation and oxidative stress. Trehalose’s unique structure, featuring a non-reducing terminal hydroxyl group, prevents it from participating in glycation reactions, making it particularly effective in inhibiting AGE formation when interacting with proteins. Additionally, trehalose enhances the intrinsic antioxidant properties of HA, providing a more robust defense against reactive oxygen species that are typically elevated during glycation processes. This enhanced antioxidative capacity increases the content of endogenous antioxidants and effectively scavenges hydrogen peroxide and superoxide anions, thereby interrupting the oxidative stress cycle that accelerates AGE formation. The HA-trehalose complex further preserves the mechanical and functional properties of structural proteins within the skin matrix, while the autophagy-activating properties of trehalose may contribute to the clearance of glycated proteins. These molecular interactions explain how the protective product maintained CML levels close to baseline despite methylglyoxal-induced glycation stress in our experimental model [1,4,5,6,7,8,9].

When examining the response to methylglyoxal-induced glycation stress, the Pp maintained its effectiveness, resulting in a 30% reduction in CML formation compared to methylglyoxal-treated samples. Importantly, explants treated with both Pp and methylglyoxal maintained CML levels comparable to baseline conditions, with no significant difference observed between PMGp and initial conditions (*p* = 1.000) or day 6 controls (*p* = 1.000).

This maintenance of baseline-like conditions under glycation stress aligns with the mechanisms of the HA-trehalose complex, which preserves the mechanical and functional properties of structural proteins within the skin matrix while providing enhanced defense against reactive oxygen species and external stressors [12,13,14].

An interesting observation emerged regarding the baseline levels of CML in the tissue. The product reduced CML formation to what appears to be a physiological baseline, suggesting the existence of a natural threshold for AGE presence in skin tissue.

To contextualize our findings within the landscape of current anti-glycation treatments, it is valuable to compare our results with other recently studied products. For instance, Havas et al. [15] demonstrated that a Dunaliella salina extract reduced CML formation by up to 68% in methylglyoxal-challenged skin explants, though this was achieved after 10 days of treatment with multiple topical applications. In comparison, our injectable HA-trehalose formulation achieved a significant 45% reduction in CML formation under baseline conditions and a 30% reduction under intense methylglyoxal-induced stress after only 6 days and with a single prophylactic administration. This efficiency is particularly relevant in the context of injectable treatments, where minimizing the frequency of applications is advantageous.

It is also informative to compare our results with other anti-glycation approaches using different active ingredients. Shin et al. [16] investigated methyl gallate, a phenolic compound with antioxidant properties, in a methylglyoxal-induced glycation model using human skin explants. Their study used the same concentration of methylglyoxal (500 μM) as our study, but applied it intermittently (on days 3, 5, and 7) rather than continuously. Under these conditions, they demonstrated that topical application of 0.1% methyl gallate completely inhibited CML expression in explants after 8 days of treatment with multiple applications. In comparison, our study used a more challenging glycation protocol with continuous methylglyoxal exposure from day 1 to day 4, which likely created a more intense glycation stress. Despite this more aggressive glycation model, our HA-trehalose formulation still achieved a 30% reduction in CML formation with just a single prophylactic administration. This distinction highlights the robust protective capability of our formulation even under severe glycation conditions, and offers the clinical advantage of requiring fewer interventions, which is particularly relevant for injectable treatments.

Our injectable formulation combines different molecular weights of hyaluronic acid with trehalose in a unique approach to anti-glycation protection. While trehalose has been studied as a stabilizing agent for HA [8], protecting it from degradation by hyaluronidase, and some research has examined HA-trehalose combinations in ophthalmology and orthopedic applications [17,18,19], our study represents the first investigation of an injectable formulation using multiple HA chain lengths with trehalose specifically for dermal anti-glycation purposes. This approach leverages the synergistic effects of different molecular weight HA fractions, each contributing distinct benefits, while trehalose enhances stability and provides additional antioxidant protection. The result is a comprehensive anti-glycation solution that addresses multiple aspects of the glycation process through a single prophylactic administration. The strength of these effects is supported by our ANOVA results, which revealed a large effect size (η^2^ = 0.56), indicating that 56% of the variance in CML levels can be attributed to our experimental treatments.

It is important to acknowledge that our study utilized skin explants from a single 28-year-old female donor with phototype III, which may limit the generalizability of our findings. While this approach controlled for biological variability, it does not account for potential variations in response across different demographic groups with varying susceptibility to glycation. Future studies should expand to include multiple donors of varying ages, genders, and skin phototypes to validate our findings across diverse populations. Despite this limitation, our controlled experimental design with standardized glycation induction provides valuable preliminary evidence for the anti-glycation potential of our formulation.

This is supported by our observation that control samples (T0) showed CML presence in 4.7% of the reticular dermis surface, with control samples at day 6 (TJ6) exhibiting similar levels at 5% of the surface area.

The product also demonstrated excellent tolerability throughout the experimental period, with no adverse effects on cell viability in either control or methylglyoxal-treated conditions. This safety profile, combined with the significant reduction in CML formation, suggests potential utility as a protective treatment against glycation-related skin aging processes.

These findings provide strong evidence for the product’s protective activity against both baseline and methylglyoxal-induced glycation in the reticular dermis of human skin explants. The ability to maintain CML levels close to baseline despite glycation stress indicates a robust protective mechanism that could be valuable in preventing glycation-related skin aging.

## 4. Materials and Methods

This study was conducted in the spirit of the Good Laboratory Practices (Arrêté du 10 Août 2004), as well as in compliance with the validated procedures and SOP of Eurofins BIO-EC.

### 4.1. Schedule of the Study

**Figure 5:**
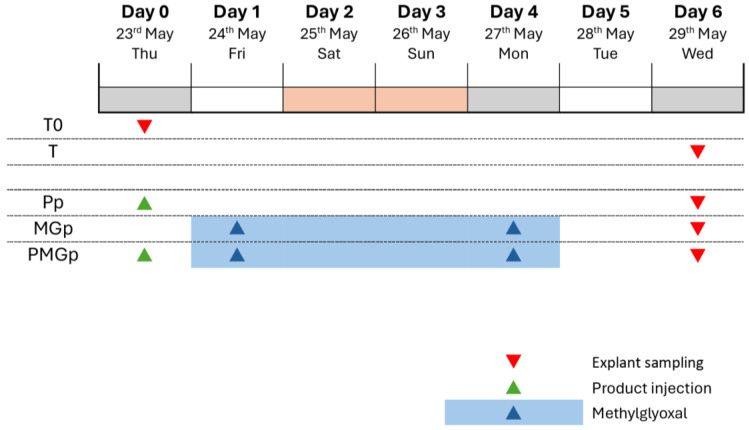
Experimental timeline for the glycation protection study. The 7-day protocol shows sampling points and intervention timepoints across different conditions.

The experimental protocol was conducted over a 6-day period, from 23 May to 29 May. Multiple experimental conditions were established to evaluate protective effects against glycation: The control series included an initial baseline (T0) sampled on day 0, followed by time-matched controls (T) sampled on day 6. For the protective effect evaluation (Pp), the product was administered on day 0, prior to methylglyoxal exposure. In the protective glycation model (MGp), methylglyoxal treatment was applied from day 1 to day 4, with sampling conducted on day 6 (Figure 5). This experimental design allowed for the assessment of preventive interventions against glycation-induced changes in the skin explants.

### 4.2. Tested Product

Twenty-one human skin explants of an average diameter of 11 mm (±1 mm) were prepared on an abdominoplasty coming from a 28-year-old Caucasian woman (reference: P2980-AB28) with a phototype III based on Fitzpatrick skin color classification. On day 0, the explants were kept in survival in BEM culture medium (BIO-EC’s Explants Medium) at 37 °C in a humid, 5%-CO_2_ atmosphere. This study is performed on skin tissue obtained from surgical residues (aesthetic surgery) of one donor in full respect of the Declaration of Helsinki and the article L.1243-4 of the French Public Health Code. The latter does not require any prior authorization by an ethics committee for sampling and using surgical wastes.

### 4.3. Explant Distribution

The study design included control groups and protective effect groups. For control conditions, three explants were allocated to baseline tissue control (T0) sampled on day 0, while nine explants were assigned to non-treated control groups (T), with three samples each collected on day 6 (Table 3).

For the protective effect assessment, nine explants were distributed across three experimental conditions, with three explants per group. These included a group treated with the injectable product P (Pp), a methylglyoxal-treated group (MGpT), and a combined treatment group receiving both injectable product P and methylglyoxal (PMGp). All protective effect groups were sampled on day 6 of the experiment (Table 4).

This distribution scheme enabled the evaluation of both baseline tissue conditions and the protective effects of the test product against methylglyoxal-induced changes in the skin explants.

### 4.4. Product Treatment

For the protective effect (batches Pp and PMGp), an intra-dermal injection of the protective product Pp (Resteeme X; Distributed by URGO Sp. z o. o., Warsaw, Poland) (30 µL) was performed on day 0 (1 day before glycation induction) using the syringe provided by the sponsor of the study. The product was brought to room temperature before injection. The control batches “T” did not receive any treatment except the renewal of the culture medium. Half of the culture (1 mL) medium was refreshed on D1 and D4 and completely renewed (2 mL) on D5.

### 4.5. Induction of Glycation

To induce the glycation on all “MG” batches, the methylglyoxal solution was incorporated in the BEM culture medium at a final concentration of 500 µM on day 1 and day 4. On day 5, the whole culture medium of the batch “MG” was replaced by 2 mL of fresh BEM culture medium without methylglyoxal and then renewed without methylglyoxal.

### 4.6. Sampling

On D0, the 3 explants from the batch T0 were collected and cut into two parts. Half was fixed in buffered formalin solution and half was frozen at −80 °C. On D6, for all batches except MGc, 3 explants were collected and treated in the same way as in D0. According to the dispositions mentioned in the study plan, the days of treatments were adjusted to the schedule of working days.

### 4.7. Histological Processing

After fixation for 24 h in buffered formalin, the samples were dehydrated and impregnated in paraffin using a Leica PEARL dehydration automat. The samples were embedded using a Leica EG 1160 embedding station. Five-micrometer-thick sections were made using a Leica RM 2125 Minot-type microtome, and the sections were mounted on Superfrost^®^ histological glass slides. The microscopical observations were realized using a Leica DMLB, an Olympus BX43 or BX63 microscope. Pictures were digitized with a numeric DP Olympus camera with Olympus cellsens 4.3 software (Olympus Evident, Rungis, France).

#### 4.7.1. Viability Control

The cell viability of the epidermal and dermal structures was controlled on formalin-fixed paraffin-embedded (FFPE) skin sections after Masson’s trichrome staining, Goldner variant. The cell viability was assessed by microscopical observation.

#### 4.7.2. CML Immunostaining

CML immunostaining has been achieved on FFPE skin sections with a monoclonal anti-CML antibody (TransGenic ref. KH011, clone CMS-10) diluted at 1:50 in PBS-BSA 0.3% overnight at room temperature using a Vectastain Kit Vector amplifier system avidin/biotin, and revealed by VIP (Vector, ref. PK-7200), a substrate of peroxidase giving a violet staining once oxidized. The immunostaining was assessed by microscopical observation and semi-quantified by image analysis using the software cellSens (Olympus).

Analysed zone: ROI including the reticular dermis. Number of analysed images per batch: 9.

### 4.8. Image Analysis

The image analyses were performed on all the images of each batch, according to the following method using cellSens software (Olympus).

**Figure 6:**
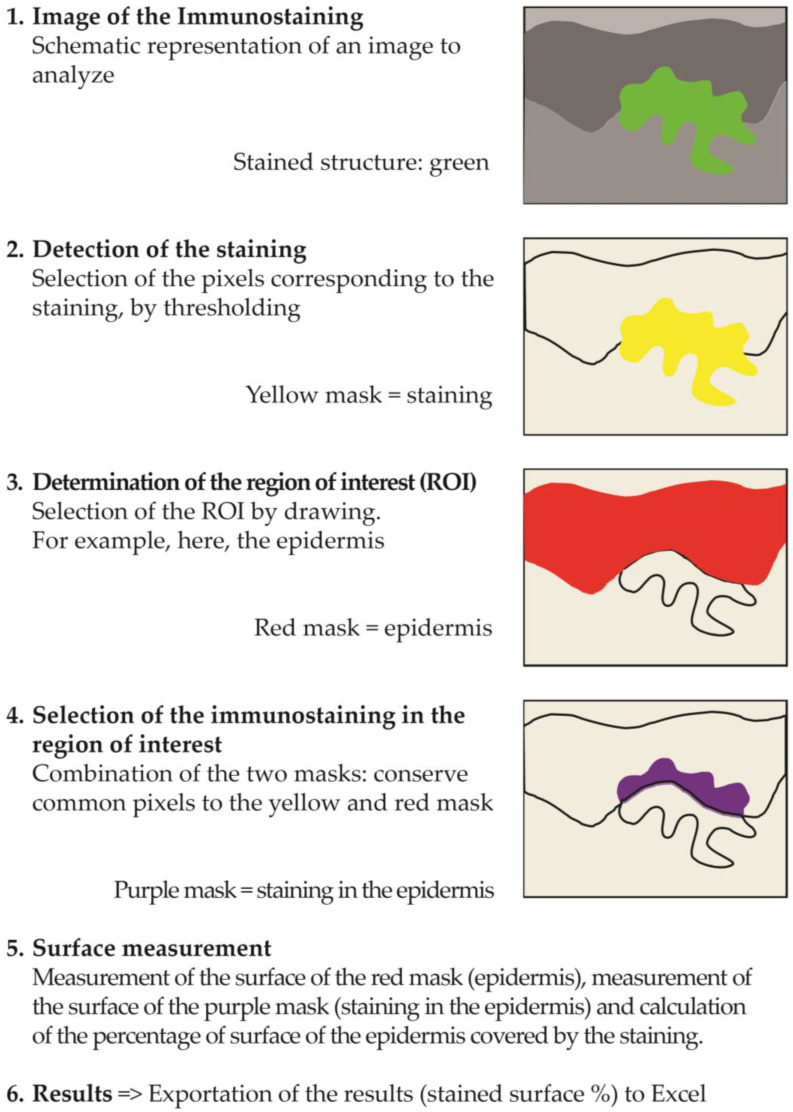
Step-by-step illustration of the image analysis process used to quantify CML immunostaining in the epidermis and reticular dermis of human skin explants.

The image analysis process followed a systematic six-step procedure for quantifying immunostaining in tissue samples (Figure 6). Initially, images of the immunostained structures were captured, appearing as green staining in the microscopic field. The second step involved defining the region of interest (ROI) by manually drawing a boundary around the target area (such as the epidermis), which was represented by a red mask. In the third step, the immunostaining was detected by thresholding to select pixels corresponding to the staining, resulting in a yellow mask. The fourth step combined the ROI and staining masks to identify the immunostaining specifically within the region of interest, visualized as a purple mask representing the overlap between the yellow staining and red ROI masks. Surface measurements were then performed in the fifth step, calculating both the total area of the ROI and the area of immunostaining within it, allowing for the determination of the percentage of ROI surface covered by the staining. Finally, these quantitative results were exported to Excel for further analysis.

The quantitative analysis was performed by measuring the percentage of stained surface area within the defined region of interest for each experimental batch. This measurement was conducted using the previously described image analysis process. Comparative analyses were then performed between different experimental conditions, specifically evaluating the stained surface percentages (Surf%) between methylglyoxal-treated samples and either untreated controls or product-treated samples (e.g., comparing MG vs. T or MG vs. PMG). This analytical approach allowed for the quantitative assessment of treatment effects on the immunostained parameters of interest.

### 4.9. Statistical Analysis

Statistical analysis was performed using repeated measures analysis of variance. A post hoc Bonferroni test was used to compare between the individual measurements. The difference between two batches is significant if *p* < 0.01 (**), so a probability of 99% for two batches to be significantly different or *p* < 0.05 (*), so a probability of 95% for two batches to be significantly different * significant with *p* < 0.05 (95%), ** significant with *p* < 0.01 (99%), NS non-significant.

## 5. Conclusions

The protective effect of an injectable product based on a combination of two different ranges of MWHA (Low and Mid/High) and trehalose against glycation was evaluated in human skin explants. The product demonstrated excellent tolerability through day 6 of the experiment, with no adverse effects on cell viability in either control or methylglyoxal-treated conditions.

When examining the protective capabilities against glycation, the product was administered one day prior to methylglyoxal-induced glycation. Quantitative analysis of CML immunostaining in the reticular dermis revealed that the product significantly reduced CML formation by 45% (*p* < 0.01) compared to untreated controls. Furthermore, in the presence of methylglyoxal-induced glycation, the product maintained its protective efficacy, resulting in a significant 30% reduction (*p* < 0.05) in CML formation compared to methylglyoxal-treated samples.

These results demonstrate that an injectable product based on a combination of two different ranges of MWHA (Low and Mid/High) and trehalose exhibits substantial protective activity against glycation when administered prophylactically, effectively preventing CML formation in the reticular dermis of human skin explants. The product’s ability to significantly reduce both baseline and methylglyoxal-induced CML formation suggests its potential utility as a preventive treatment against glycation-related skin aging processes.

## 6. Patents

“Injectable composition including hyaluronic acid and use of the said composition” US 20210315804A1.

## Figures and Tables

**Table 1:** Summary of CML immunostaining surface percentage (mean ± SD) in the reticular dermis of human skin explants under different treatment conditions. T0: initial time point; TJ6: control at day 6; PpJ6: protective product treatment at day 6; MGpJ6: methylglyoxal treatment at day 6; PMGpJ6: protective product + methylglyoxal treatment at day 6. Values represent the mean percentage of CML-positive surface area with corresponding standard deviation (SD) for each condition.

CML (% Surface)					
	T0	TJ6	PpJ6	MGpJ6	PMGpJ6
Mean	4.7	5.0	2.8	7.0	4.9
SD	1.7	0.9	0.8	2.4	1.6

**Table 2:** Statistical analysis (*p*-values) from one-way repeated measures ANOVA of CML immunostaining differences between experimental conditions in the reticular dermis. T0: initial time point; TJ6: control at day 6; PpJ6: protective product treatment at day 6; MGpJ6: methylglyoxal treatment at day 6; PMGpJ6: protective product + methylglyoxal treatment at day 6. * significant with *p* < 0.05 (95%); ** significant with *p* < 0.01 (99%).

	T0	TJ6	PpJ6	MGpJ6	PMGpJ6
**T0**		1.000	0.035 *	0.306	1.000
**TJ6**	1.000		0.008 **	0.173	1.000
**PpJ6**	0.035 *	0.008 **		0.020 *	0.026 *
**MGpJ6**	0.306	0.173	0.020 *		0.507
**PMGpJ6**	1.000	1.000	0.026 *	0.507	

**Table 3:** Distribution of human skin explants for control conditions showing batch type (T0: tissue control; T: non-treated control), experimental conditions, number of explants per group (n = 3), and sampling time points.

Control (Untreated) Condition			
Batch	Conditions	Nb of Explants	Sampling Time
**T0**	Tissue control	3	Day 0
**T**	Non-treated control	3, 3, 3	Day 6

**Table 4:** Distribution of human skin explants for protective effect assessment showing batch types (Pp: injectable product P; MGp: methylglyoxal; PMGp: injectable product P + methylglyoxal), experimental conditions, number of explants per group (n = 3), and sampling time points.

Protective Effect			
Batch	Conditions	Nb of Explants	Sampling Time
**Pp**	Injectable product P	3	Day 6
**MGp**	Methylglyoxal	3	Day 6
**PMGp**	Injectable product P + Methylglyoxal	3	Day 6

## Data Availability

The datasets used and/or analyzed during the current study are available from the corresponding author on reasonable request.

## Abbreviations

The following abbreviations are used in this manuscript:

MWHA	Molecular Weight of Hyaluronic Acid
LMWHA	Low Molecular Weight of Hyaluronic Acid
MMWHA	Mid Molecular Weight of Hyaluronic Acid
HMWHA	High Molecular Weight of Hyaluronic Acid
AGEs	Advanced Glycation End-products
CML	Carboxymethyllysine
HA	Hyaluronic acid
NF-κB	Nuclear factor kappa-light-chain-enhancer of activated B cells
TNF-α	Tumor necrosis factor-α
Pp	protective product
TJ6	control samples at day 6
T0	initial time point
PpJ6	protective product treatment at day 6
MGpJ6	glycation-inducing stressor methylglyoxal at day 6
PMGpJ6	combined treatment with protective product and glycation-inducing methylglyoxal at day 6
ROI	region of interest

## References

[1] Chmielewski R., Lesiak A. (2024). Mitigating Glycation and Oxidative Stress in Aesthetic Medicine: Hyaluronic Acid and Trehalose Synergy for Anti-AGEs Action in Skin Aging Treatment. Clin. Cosmet. Investig. Dermatol..

[2] Thorpe S.R., Baynes J.W. (2003). Maillard reaction products in tissue proteins: New products and new perspectives. Amino Acids.

[3] Gkogkolou P., Böhm M. (2012). Advanced glycation end products: Key players in skin aging?. Derm.-Endocrinol..

[4] Maria-Engler S.S. (2017). Assessing the effects of advanced glycation end products in the skin. Br. J. Dermatol..

[5] Wu H., Chen H., Zheng Z., Li J., Ding J., Huang Z., Jia C., Shen Z., Bao G., Wu L. (2019). Trehalose promotes the survival of random-pattern skin flaps by TFEB mediated autophagy enhancement. Cell Death Dis..

[6] Li L., Chen H., Chen X., Chen S., Gu H. (2022). Trehalose protects keratinocytes against ultraviolet B radiation by activating autophagy via regulating TIMP3 and ATG9A. Oxidative Med. Cell. Longev..

[7] Yin L., Wei-Min L., Wei W. (2008). Trehalose: Protector of antioxidant enzymes or reactive oxygen species scavenger under heat stress?. Environ. Exp. Bot..

[8] Morales M., Gobbi A. (2021). Trehalose: A Promising Stabilizer Agent for Hyaluronic Acid.

[9] Zheng W., Li H., Go Y., Chan X.H., Huang Q., Wu J. (2022). Research advances on the damage mechanism of skin glycation and related inhibitors. Nutrients.

[10] Sugiura K., Koike S., Suzuki T., Ogasawara Y. (2021). Carbonylation of skin collagen induced by reaction with methylglyoxal. Biochem. Biophys. Res. Commun..

[11] Lai S.W.T., Lopez Gonzalez E.D.J., Zoukari T., Ki P., Shuck S.C. (2022). Methylglyoxal and its adducts: Induction, repair, and association with disease. Chem. Res. Toxicol..

[12] Yung S., Chan T.M. (2011). Pathophysiology of the peritoneal membrane during peritoneal dialysis: The role of hyaluronan. BioMed Res. Int..

[13] Mongkhon J.M., Thach M., Shi Q., Fernandes J.C., Fahmi H., Benderdour M. (2014). Sorbitol-modified hyaluronic acid reduces oxidative stress, apoptosis and mediators of inflammation and catabolism in human osteoarthritic chondrocytes. Inflamm. Res..

[14] Juncan A.M., Moisă D.G., Santini A., Morgovan C., Rus L.-L., Vonica-Țincu A.L., Loghin F. (2021). Advantages of hyaluronic acid and its combination with other bioactive ingredients in cosmeceuticals. Molecules.

[15] Havas F., Krispin S., Cohen M., Loing E., Farge M., Suere T., Attia-Vigneau J. (2022). A Dunaliella salina extract counteracts skin aging under intense solar irradiation thanks to its antiglycation and anti-inflammatory properties. Mar. Drugs.

[16] Shin S., Lee J., Yoon S.H., Park D., Hwang J.S., Jung E. (2022). Anti-glycation activities of methyl gallate in vitro and in human explants. J. Cosmet. Dermatol..

[17] Gobbi A., Morales M., Avio G., D’Ambrosi R. (2022). Double-blinded prospective randomized clinical trial in knee joint osteoarthritis treatment: Safety assessment and performance of trehalose hyaluronic acid versus standard infiltrative therapy based on medium-weight sodium hyaluronate. J. Cartil. Jt. Preserv..

[18] Mencucci R., Favuzza E., Decandia G., Cennamo M., Giansanti F. (2021). Hyaluronic acid/trehalose ophthalmic solution in reducing post-cataract surgery dry eye signs and symptoms: A prospective, interventional, randomized, open-label study. J. Clin. Med..

[19] Fariselli C., Giannaccare G., Fresina M., Versura P. (2018). Trehalose/hyaluronate eyedrop effects on ocular surface inflammatory markers and mucin expression in dry eye patients. Clin. Ophthalmol..

